# Supplementary material for: Interferon-γ increases sensitivity to chemotherapy and provides immunotherapy targets in models of metastatic castration-resistant prostate cancer
Source: Sci Rep. 2022 Apr 22;12:6657. doi: 10.1038/s41598-022-10724-9 (PMC9033763; doi:10.1038/s41598-022-10724-9)
Supplement: Supplementary file 1 — Supplementary Information. [file 41598_2022_10724_MOESM1_ESM.pdf]

SUPPLEMENTARY INFORMATION for:

Interferon-γ Increases Sensitivity to Chemotherapy and Provides Immunotherapy Targets in Models of Metastatic Castration-Resistant Prostate Cancer

Dimitrios Korentzelos [1], Alan Wells [1,2,3,4,5,6] and Amanda M. Clark [1,2,3,6] *

[1] Department of Pathology, University of Pittsburgh, Pittsburgh, PA 15261, USA
[2] UPMC Hillman Cancer Center, University of Pittsburgh, Pittsburgh, PA 15213, USA
[3] VA Pittsburgh Healthcare System, Pittsburgh, PA 15213, USA
[4] Department of Bioengineering, University of Pittsburgh, Pittsburgh, PA 15260, USA
[5] Department of Computational & Systems Biology, University of Pittsburgh, Pittsburgh, PA 15260, USA
[6] Pittsburgh Liver Research Center, University of Pittsburgh and UPMC, Pittsburgh, PA 15261, USA
* Correspondence: Amanda M. Clark, amc235@pitt.edu

Figures:

Fig S1 In vitro dose optimization of IFNγ.
Fig S2. Uncropped immunoblots of Figure 1B.
Fig S3. IFNγ induces upregulation of MHC-I and PD-L1, and downregulation of E-cadherin in benign prostate epithelial cells.
Fig S4. Effects of IFNγ on membranous MHC-I, PD-L1, and E-cadherin expression in DU145 cells.
Fig S5. IFNγ induces upregulation of MHC-I and PD-L1, and downregulation of E-cadherin in PC3-H cells.
Fig S6. IFNγ induces upregulation of MHC-I and PD-L1, and downregulation of E-cadherin in PC3-L cells.
Fig S7. Effects of IFNγ on MHC-I, PD-L1 and E-cadherin expression in LnCaP cells.
Fig S8. IFNγ influences the response of mCRPC cells to paclitaxel in vitro.
Fig S9. Effects on HLA-A, PD-L1 and E-cadherin expression and induction of chemosensitivity after IFNγ pretreatment in vivo.

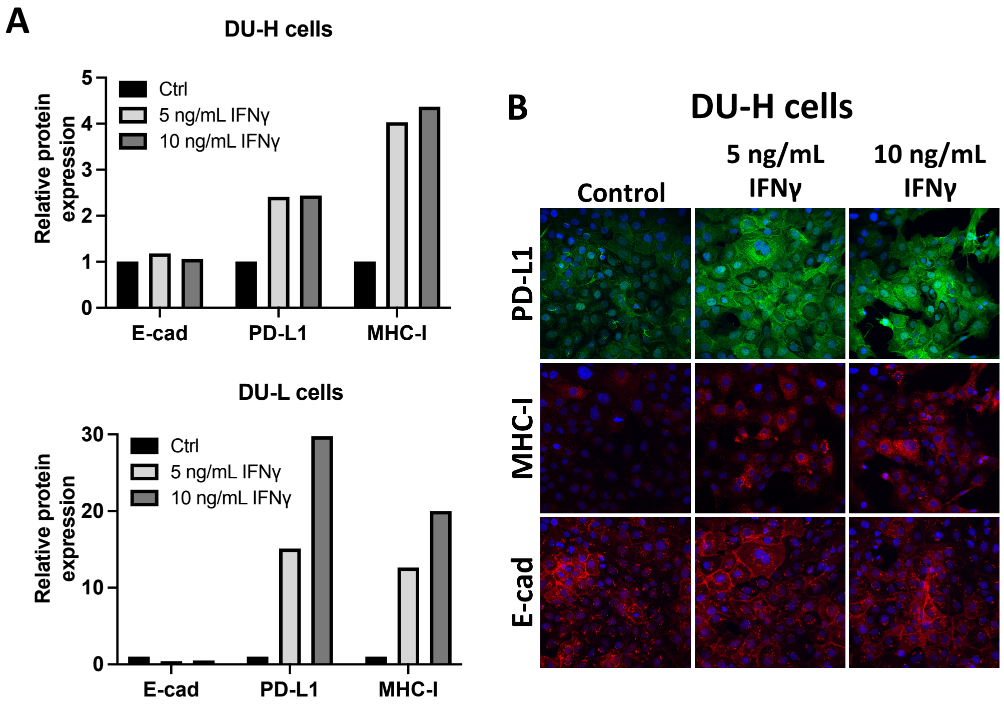

Fig. S1. In vitro dose optimization of IFNγ. (A) Quantification of immunoblots of E-cadherin, PD-L1, and MHC-I in DU-H and DU-L cells after control or IFNγ (5 ng/mL) treatment for 48 hours, with GAPDH as loading control. (B) Representative immunofluorescence images of PD-L1 (green), MHC-I (red), E-cadherin (red), and Hoechst 33342 (blue) in DU-H cells.

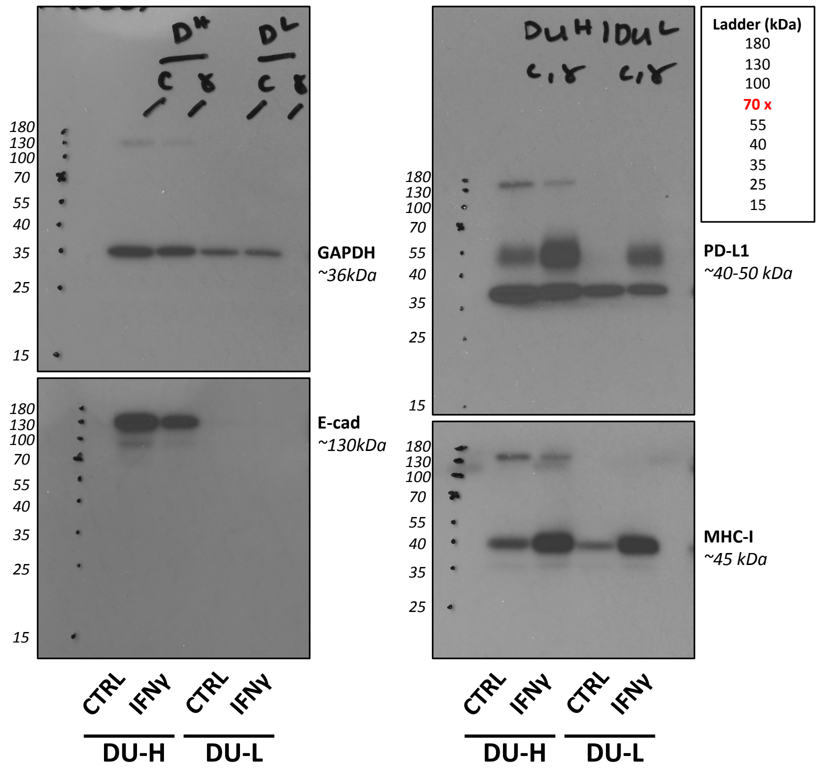

Fig. S2. Uncropped immunoblots of Figure 1B. Immunoblots of E-cadherin, PD-L1, and MHC-I for DU-H and DU-L cells after control or IFNγ (5 ng/mL) treatment for 48 hours, with GAPDH as loading control.

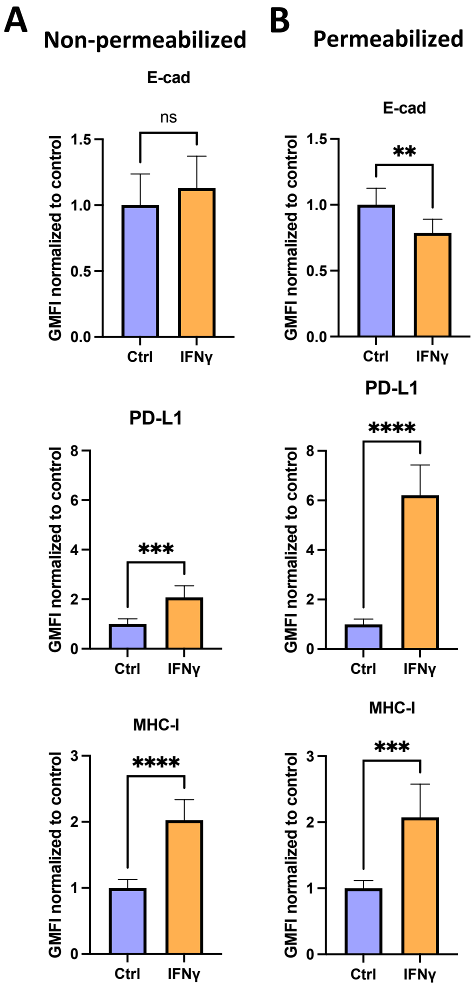

Fig. S3. IFNγ induces upregulation of MHC-I and PD-L1, and downregulation of E-cadherin in benign prostate epithelial cells. Geometric Mean Fluorescence Intensity (GMFI) of E-cadherin, MHC-I, and PD-L1 membranous (non-permeabilized cells) and total (permeabilized cells) expression in RWPE cells after treatment with control or IFNγ (5 ng/mL) for 48 hours, determined by flow cytometry. Data shown as mean ± SD. Student t-test, ns, not significant, **$p<0.005$, ***$p<0.001$, ****$p<0.0001$.

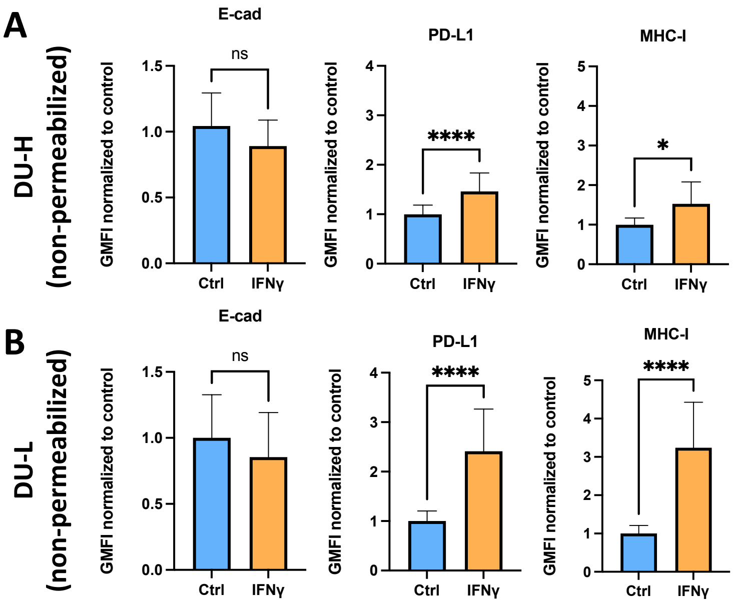

Fig. S4. Effects of IFNγ on membranous MHC-I, PD-L1 and E-cadherin expression in DU145 cells. GMFI of membranous E-cadherin, MHC-I, and PD-L1 expression in DU-H and DU-L (non-permeabilized) prostate cancer cells after treatment with control or IFNγ (5 ng/mL) for 48 hours, determined by flow cytometry. Data shown as mean ± SD of at least three independent experiments. Student t-test, ns, not significant, *p<0.05, ****p<0.0001.

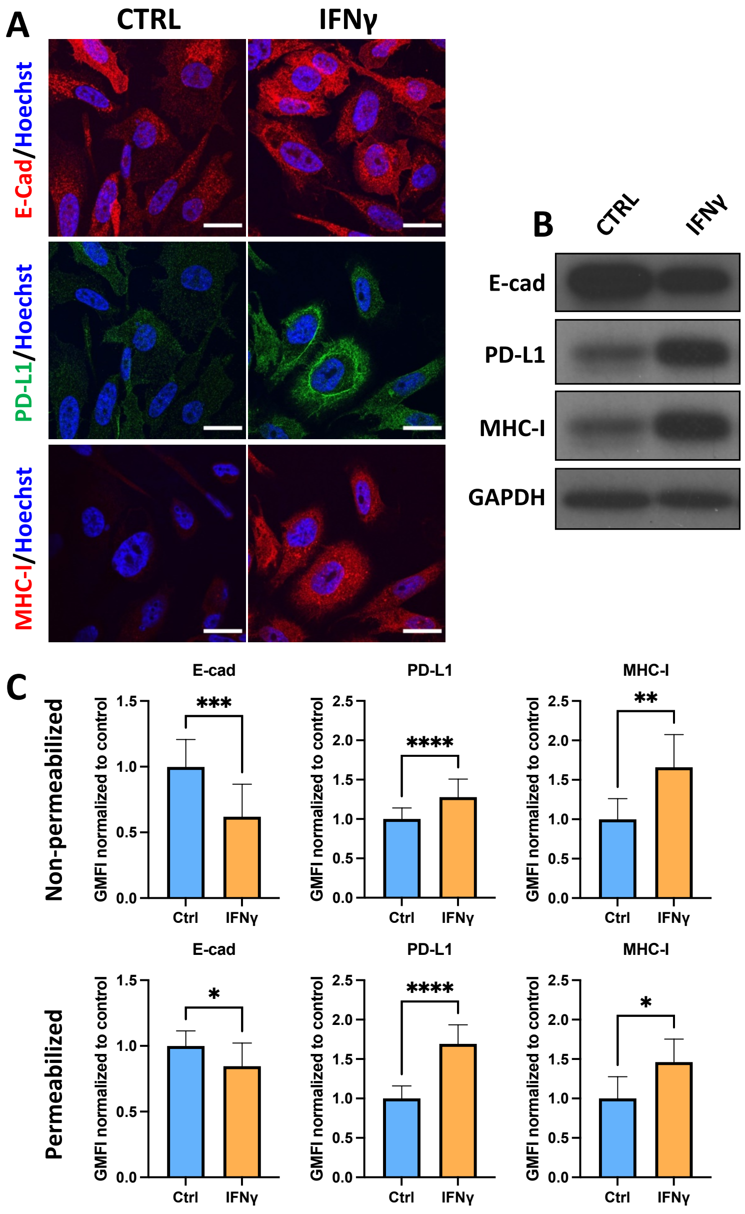

Fig. S5. IFNγ induces upregulation of MHC-I and PD-L1, and downregulation of E-cadherin in PC3-H cells. (A) Representative immunofluorescence images of staining MHC-I (red), PD-L1 (green), E-cadherin (red), and Hoechst 33342 (blue) in PC3-H cells. Cells were treated with control or IFNγ (5 ng/mL) for 48 hours. All scale bars, 50 µm. (B) Western blot of E-cadherin, PD-L1, and MHC-I in DU-H and DU-L cells after control or IFNγ (5 ng/mL) treatment for 48 hours, with GAPDH as loading control. (C) GMFI of E-cadherin, MHC-I, and PD-L1 membranous (non-permeabilized cells) and total (permeabilized cells) expression in PC3-H cells after treatment with control or IFNγ (5 ng/mL) for 48 hours, determined by flow cytometry. Data shown as mean ± SD of at least three independent experiments. Student t-test, *p<0.05, **p<0.005, ***, p<0.001, ****p<0.0001.

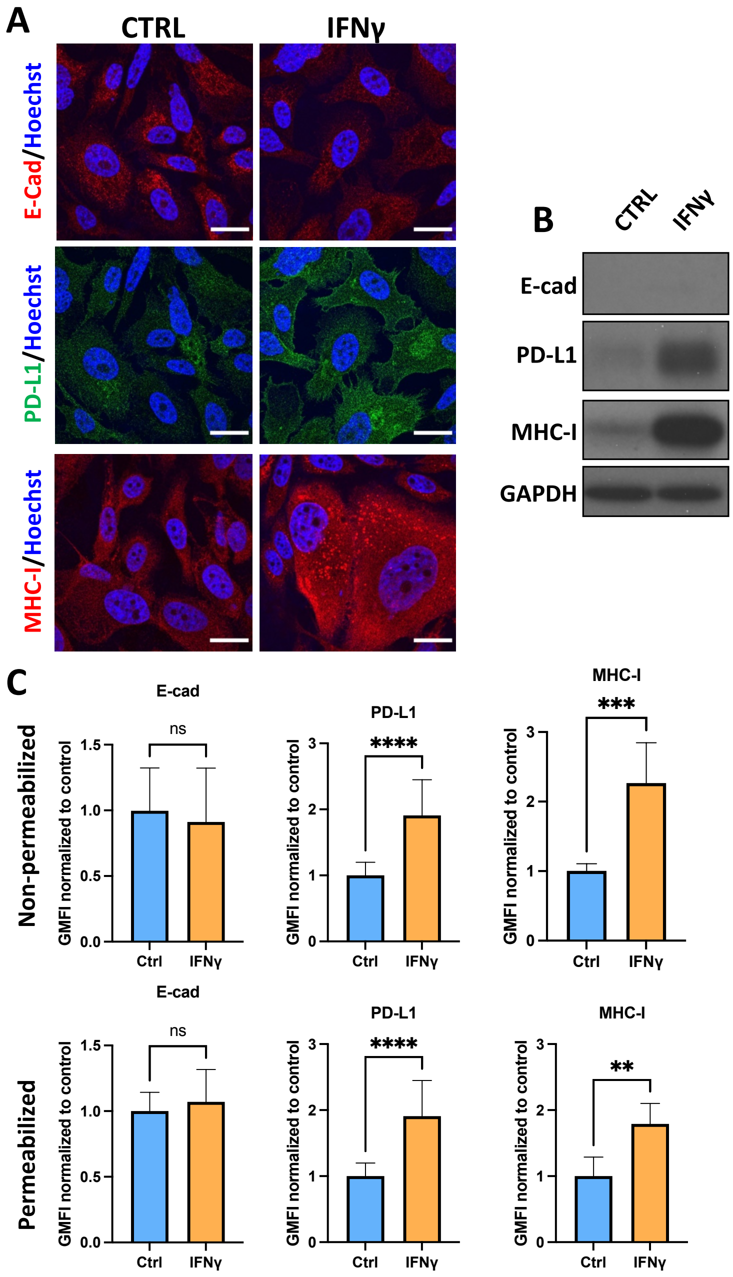

Fig. S6. IFNγ induces upregulation of MHC-I and PD-L1, and downregulation of E-cadherin in PC3-L cells. (A) Representative immunofluorescence images of staining MHC-I (red), PD-L1 (green), E-cadherin (red), and Hoechst 33342 (blue) in PC3-L cells. Cells were treated with control or IFNγ (5 ng/mL) for 48 hours. All scale bars, 50 µm. (B) Western blot of E-cadherin, PD-L1, and MHC-I in DU-H and DU-L cells after control or IFNγ (5 ng/mL) treatment for 48 hours, with GAPDH as loading control. (C) GMFI of E-cadherin, MHC-I, and PD-L1 membranous (non-permeabilized cells) and total (permeabilized cells) expression in PC3-L cells after treatment with control or IFNγ (5 ng/mL) for 48 hours, determined by flow cytometry. Data shown as mean ± SD of at least three independent experiments. Student t-test, ns, not significant, **p<0.005, ***, p<0.001, ****p<0.0001.

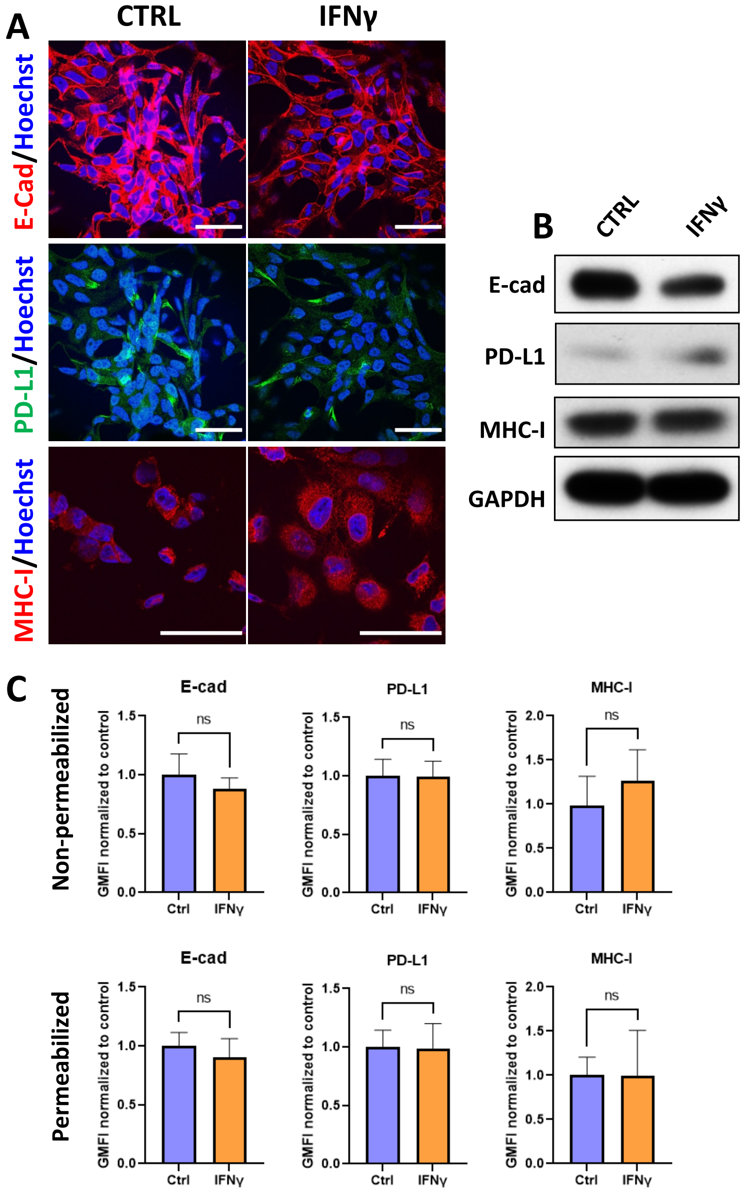

Fig. S7. Effects of IFNγ on MHC-I, PD-L1 and E-cadherin expression in LnCaP cells. (A) Representative immunofluorescence images of staining MHC-I (red), PD-L1 (green), E-cadherin (red), and Hoechst 33342 (blue) in LnCaP cells. Cells were treated with control or IFNγ (5 ng/mL) for 48 hours. All scale bars, 50 µm. (B) Western blot of E-cadherin, PD-L1, and MHC-I after control or IFNγ (5 ng/mL) treatment for 48 hours, with GAPDH as loading control. (C) GMFI of E-cadherin, MHC-I, and PD-L1 membranous (non-permeabilized) and total (permeabilized) expression in LnCaP cells after treatment with control or IFNγ (5 ng/mL) for 48 hours, determined by flow cytometry. Data shown as mean±SD of three independent experiments. Student t-test, ns, not significant.

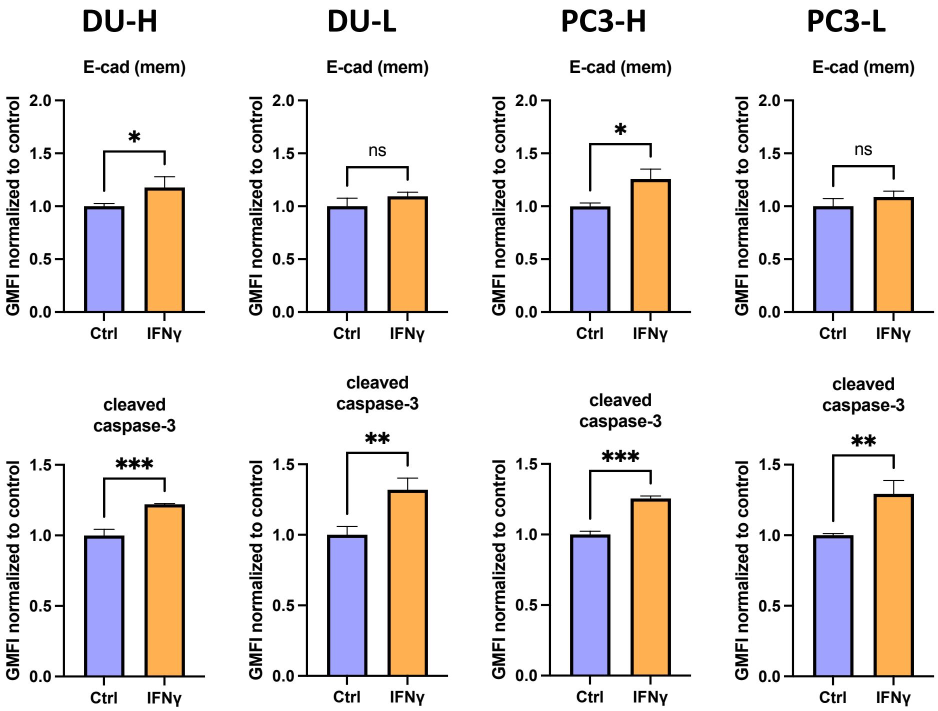

Fig S8. IFNγ influences the response of mCRPC cells to paclitaxel in vitro. GMFI of cleaved caspase 3 and membranous E-cadherin expression in DU-H, DU-L, PC3-H and PC3-L cells determined by flow cytometry. Cells were treated with 1 µM paclitaxel and 100 ng/ml TRAIL (PAC-TRAIL) for 4 hours after 48 hours of control or IFNγ (5 ng/mL) treatment. Data shown as mean ± SD. Student t-test, *$p<0.05$, **$p<0.005$, ***, $p<0.001$,

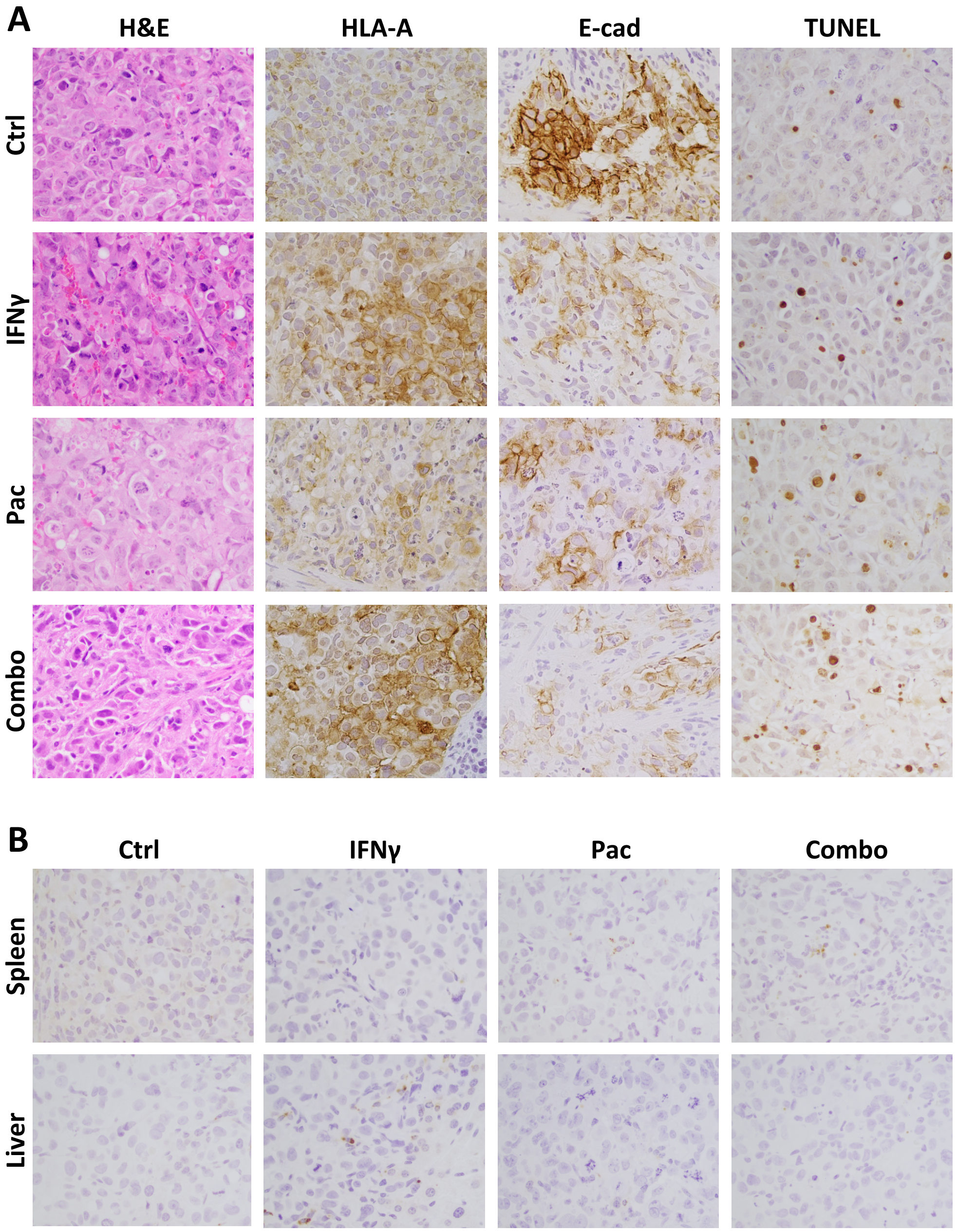

Fig. S9. Effects on HLA-A, PD-L1 and E-cadherin expression and induction of chemosensitivity after IFNγ pretreatment in vivo. (A) Representative H&E, HLA-A, E-cadherin and TUNEL staining in the primary (splenic) tumors at completion of the study. (B) Representative PD-L1 staining in primary (splenic) and metastatic (liver) setting at completion of the study. All images at 400x magnification. Tumor is not outlined where the vast majority of the captured image is tumor.
